# Genomic variation drives plant flavor diversification

**DOI:** 10.1111/jipb.70283

**Published:** 2026-05-10

**Authors:** Huimin Hu, Yanwei Hao, Yiwei Zhou, Xia Zhang, Rui Xia, Pan Liao

**Affiliations:** ^1^ Department of Biology Hong Kong Baptist University Hong Kong 999077 China; ^2^ Key Laboratory of Biology and Germplasm Enhancement of Horticultural Crops in South China Ministry of Agriculture and Rural Affairs, College of Horticulture South China Agricultural University Guangzhou 510642 China; ^3^ Guangdong Provincial Key Laboratory of Ornamental Plant Germplasm Innovation and Utilization, Environmental Horticulture Research Institute Guangdong Academy of Agricultural Sciences Guangzhou 510640 China; ^4^ State Key Laboratory of Agrobiotechnology (CUHK) Hong Kong 999077 China; ^5^ Institute of Systems Medicine and Health Sciences Hong Kong Baptist University Hong Kong 999077 China

**Keywords:** aroma, artificial intelligence, genomic variation, plant flavor, sugar and acid

## Abstract

Plant flavor diversity arises from genomic variation across species and cultivars, yet the mechanisms linking natural genomic variation to flavor phenotypes remain insufficiently integrated. Here, we systematically review how diverse forms of genomic variation reshape the biosynthesis, transport, and accumulation of flavor metabolites, thereby driving diversification in sweetness, acidity, bitterness, piquancy, astringency, and aroma. We further integrate evidence from genomics, transcriptomics, metabolomics, and functional validation to outline a mechanistic framework linking genomic variation to the molecular and metabolic basis of flavor phenotypes. We then examine how artificial intelligence‐assisted breeding and precision gene editing can accelerate the identification of causal variants and enable targeted improvement of flavor traits. This framework establishes plant flavor as a mechanism‐based target for plant improvement, with broad implications for quality, nutrition, and sustainability.

## Graphical abstract

This review explains how genomic variation shapes plant flavor by altering the biosynthetic and regulatory pathways of key attributes like sweetness, acidity, bitterness, piquancy, astringency, and aroma. It also discusses how multi‐omics, AI‐assisted breeding, and gene editing can translate this knowledge into plants with improved flavor, nutrition, and sustainability.

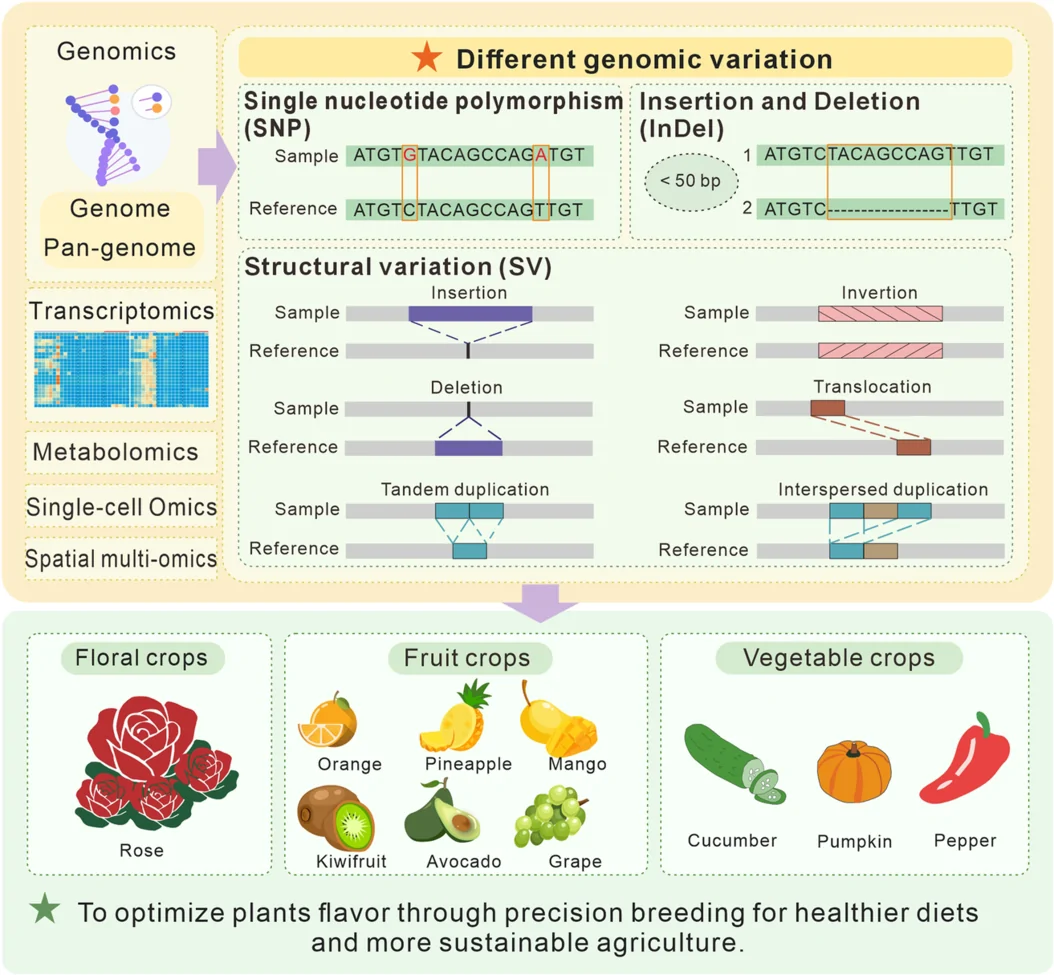

## INTRODUCTION

Plant flavor plays a critical role in promoting healthy human diets (Chen et al., 2021; Xing et al., 2025). A pleasant flavor experience serves as a direct driver for increased fruit and vegetable consumption, thereby enhancing the intake of essential health‐promoting components such as vitamins, dietary fiber, and phytochemicals (Duan et al., 2023; Chan et al., 2024; Martin and Butelli, 2024). Furthermore, flavor‐related compounds represent a chemical language that plants employ to communicate with the environment (Turlings and Erb, 2018; Stirling et al., 2024; Jin et al., 2025). For instance, the complex mixture of volatile organic compounds (VOCs) released by flowers attracts specific pollinators, ensuring precise pollination services (Boachon et al., 2019; Zhou et al., 2024). Similarly, capsaicin activates mammalian pain receptors to produce a pungent sensation, effectively deterring herbivory (Zheng et al., 2023). Remarkably, when plants are attacked by herbivorous insects, they immediately release volatile signals, such as jasmonic acid (JA) and methyl‐salicylate (MeSA), which can activate systemic defense responses (Cao et al., 2022; Gong et al., 2023). Neighboring plants that perceive this ‘alarm’ can preemptively activate defense‐related gene expression, thereby effectively deterring insect feeding (Liu et al., 2025; Luo et al., 2025). Collectively, these examples demonstrate that flavor‐related compounds not only function as core indicators of plant quality, directly influencing crop market acceptance and consumer preference, but also serve as crucial informational mediators in plant responses to biotic stress and interspecies interactions, ultimately shaping their survival, competitiveness, and reproduction within ecosystems.

The metabolic basis of plant flavor comprises three interacting components: Taste substances, aromatic compounds, and texture‐related attributes, all of which contribute to plant quality and change dynamically during development (Xue et al., 2025a). Taste substances mainly include sugars, such as fructose and glucose, which contribute to sweetness; organic acids, including malic and citric acids, which impart acidity; and free amino acids, which contribute to umami perception. These non‐volatile compounds form the core of taste perception. Aromatic compounds, particularly volatile organic compounds (VOCs), represent another major component of plant flavor profiles (Liao et al., 2023; Cao et al., 2024). To date, thousands of VOCs have been identified across various plants, such as terpenoids, esters, aldehydes, and lactones (Dudareva et al., 2013; Liu et al., 2022). Together, these compounds create distinctive aromatic fingerprints that are perceived through orthonasal and retronasal olfactory pathways (Burdach and Doty, 1987; Han et al., 2023). In addition to sugars, acids, and VOCs, plants also produce a range of secondary metabolites that contribute to bitterness, astringency, pungency, and related sensory attributes. Flavonoids are often associated with bitterness and astringency (Zhao et al., 2021), alkaloids generally yield bitter or pungent sensations (Jiang et al., 2024; Bai et al., 2025), and tannins represent the primary source of astringency in plants (Liu et al., 2023). For instance, the characteristic bitterness and lingering sweet aftertaste of tea polyphenols not only define its premium flavor profile but also form the chemical basis for its antioxidant activity (Dai et al., 2023). Correspondingly, the intense bitterness of berberine in *Coptis chinensis* (Xu et al., 2024) and the pungency of gingerols in ginger (Priyadarshini et al., 2025) are closely linked to their medicinal efficacy (Liu and Wu, 2016; Nafees et al., 2021). Importantly, plant flavor is not determined by any single class of compounds, but by interactions among volatile and non‐volatile components, including both synergistic and antagonistic effects (Yuan et al., 2023). For example, an appropriate sugar‐to‐acid ratio forms the core of a refreshing taste experience, while certain key aroma compounds can significantly enhance the overall flavor profile even at trace concentrations (Yu et al., 2015; Bergman and Dudareva, 2024; Hu et al., 2025). This complexity naturally raises a central question: What intrinsic differences underlie the formation of such diverse flavor profiles?

These complex flavor traits arise directly from specific combinations of metabolites. The biosynthesis of these metabolites, in turn, is governed by the expression of particular genes, and genomic variations often influence the expression of these key genes. Genomic variation refers to differences in DNA sequences between varieties or species, primarily including single nucleotide polymorphisms (SNPs), insertions and deletions (InDels), copy number variations (CNVs), and presence‐absence variations (PAVs) (Wu et al., 2023). On the one hand, when these variations occur in coding regions, they can directly modify the amino acid sequences of key enzymes, such as acyltransferases (AATs) and terpene synthases (TPSs), which catalyze the biosynthesis of flavor‐related compounds, consequently altering enzyme activity and substrate specificity (Fan et al., 2024; Liao et al., 2024). On the other hand, variations in regulatory regions may affect gene expression levels (Nieuwenhuizen et al., 2015; Wen et al., 2025). These natural DNA sequence variations ultimately determine the distinct flavor characteristics of different plants by influencing the function of key enzymes and regulatory factors in metabolic pathways. Therefore, elucidating the relationship between genomic variation and flavor quality will not only advance our understanding of plant evolution but also provide a theoretical foundation for breeding new cultivars with superior flavor and high nutritional value, ultimately contributing to human health through dietary improvement.

To date, most reviews in this field have primarily focused on flavor‐related metabolites and volatile compounds, biosynthetic pathways, or omics‐assisted quality improvement. In this review, we focus specifically on how genomic variation shapes plant flavor diversity across species and cultivars. We systematically summarize recent advances in understanding how different forms of genomic variation influence representative flavor attributes, including sweetness, acidity, bitterness, piquancy, astringency, and aroma. More importantly, we emphasize the molecular links between genomic variation, gene regulation, biosynthetic pathways, and the accumulation of flavor‐related metabolites. We further discuss how multi‐omics integration, artificial intelligence‐assisted breeding, and gene editing can facilitate the identification of causal variants and enable precise improvement of flavor traits. In this way, this review provides a distinct framework for understanding the origin of plant flavor diversity and for translating that knowledge into next‐generation agricultural, food, and health‐related applications.

## AN UNPRECEDENTED PACE IN UNCOVERING DIVERSE TYPES OF GENOMIC VARIATION

Genomic variation represents a fundamental source of diversity among individuals, playing a crucial role in shaping phenotypic variation and adaptive evolution in plants (Zhang et al., 2024b). These variations are broadly classified into SNPs, InDels, and structural variations (SVs) (Torkamaneh et al., 2018). SNPs, the most prevalent type of genetic variation, refer to single‐base variants, insertions, or deletions in the genome (Edwards et al., 2009; Wu et al., 2023). In contrast, InDels represent the insertion or deletion of short DNA fragments typically under 50 bp in length (Wenger et al., 2019). Unlike SNPs, InDels involve sequence fragments rather than single bases and represent the second most frequent form of genomic variation. They frequently occur in functionally important regions, such as coding sequences, exons, and promoters, often leading to altered gene function (Barton and Zeng, 2018). In addition, SVs constitute another major source of genetic diversity and generally have stronger effects on the genome than SNPs. They contribute significantly to phenotypic variation and environmental adaptation in plants. SVs encompass diverse types, including insertions and deletions larger than 50 bp, CNVs, PAVs, intra‐ and inter‐chromosomal translocations, and other complex rearrangements (Yuan et al., 2021). CNVs, typically ranging from 1 kb to 3 Mb, involve duplications, losses, inversions, or translocations of DNA fragments, with the narrow definition referring specifically to changes in gene copy number (Lye and Purugganan, 2019). Structural variations can impact genomic stability and regulate gene expression, thereby influencing plant phenotypes (Gabur et al., 2019). Understanding the relationship between genomic variation and key agronomic traits is therefore essential for improving germplasm resources, optimizing quality, enhancing yield, and strengthening stress resistance in plants.

Advances in third‐generation sequencing technologies and the release of high‐quality reference genomes have revealed that single or limited reference genomes cannot fully represent the complete genomic sequence and genetic variation within a species (Jayakodi et al., 2020). Furthermore, the complex and repetitive nature of polyploid plant genomes poses significant challenges for large‐scale genomic variation detection, leading to reduced accuracy, high costs, and consequently impeding systematic studies of trait formation mechanisms. These limitations represent a major bottleneck in modern plant breeding (Wang et al., 2023b). The successful construction of a pan‐genome offers a promising solution for overcoming this constraint by integrating all genetic variations of a population into a unified genomic framework, thereby enabling complete and accurate genotyping in plants. The first plant pan‐genome, established in 2014 through comparative analysis of seven wild soybean genomes, identified variable genes associated with important agronomic traits and paved the way for subsequent pan‐genome research in other plants (Li et al., 2014). Currently, pan‐genome studies have expanded to numerous other plant species, including model organisms such as Arabidopsis (Lian et al., 2024), rice (Zhao et al., 2018), maize (Wang et al., 2023a), tomato (Li et al., 2023b), as well as fruit crops like apple (Wang et al., 2023c), citrus (Huang et al., 2023), and grape (Liu et al., 2024b). These studies underscore the importance of pan‐genomes in plant improvement and provide a solid foundation for in‐depth analysis of genome‐trait relationships, thereby advancing plant breeding research in areas such as flavor enhancement and yield improvement.

Recent advances in systems genetics have created new research opportunities for constructing and deciphering multi‐gene regulatory networks underlying complex traits, by integrating genetic variation data with molecular profiles from multiple omics technologies, including genomics, transcriptomics, and proteomics (Christie et al., 2017). This approach has already provided a scientific foundation for pear improvement and offers valuable insights for future genetic studies and breeding efforts in other crops (Wang et al., 2023b). Furthermore, artificial intelligence is emerging as a powerful tool for multi‐omics analysis, significantly advancing our understanding of complex trait regulation in plants. In the context of genomic variation, machine learning approaches are proving particularly valuable for predicting the functional consequences of different variant types. Although the most advanced models to date have been developed in human genetics, they offer methodological frameworks that could inform similar efforts in plant systems. For example, deep learning models such as AlphaMissense integrate structural context and evolutionary conservation to predict missense variant effects at a genome‐wide scale in humans (Cheng et al., 2023); conceptually similar strategies could be developed to classify amino acid substitutions that may impact protein function in crop species. Likewise, sequence‐based frameworks like DeepSEA learn regulatory sequence codes from chromatin profiling data, enabling prediction of noncoding variant effects with single‐nucleotide sensitivity in human genomes (Zhou and Troyanskaya, 2015). If such approaches can be successfully developed and applied to plants, they will enable more precise identification and functional validation of target genes for molecular breeding, thereby enhancing breeding efficiency. This is particularly urgent in the context of climate change and a growing global population, where enhancing the yield and quality of nutritionally critical plants is an imperative. Achieving this goal requires a multi‐pronged strategy: collecting genetically diverse germplasm resources, characterizing their genomic variations, and uncovering how these variations shape key agronomic traits during domestication. These efforts, in turn, converge on a fundamental question at the heart of plant biology and breeding: How do diverse genomic variations precisely coordinate downstream metabolic pathways to generate the vast spectrum of plant flavors?

## ADVANCES IN GENOMIC VARIATION AND PLANT SWEETNESS

Sugars play a fundamental role in plant growth, development, and quality enhancement. Their concentration, composition, and proportional distribution fundamentally determine plant sweetness and flavor profiles, thereby directly influencing consumer preference (Mishra et al., 2022; Li et al., 2025a). Beyond the diversity in sugar types and levels, more and more studies have reported on the regulation of sugar content by genomic variation (Figure 1; Table 1).

**Figure 1:**
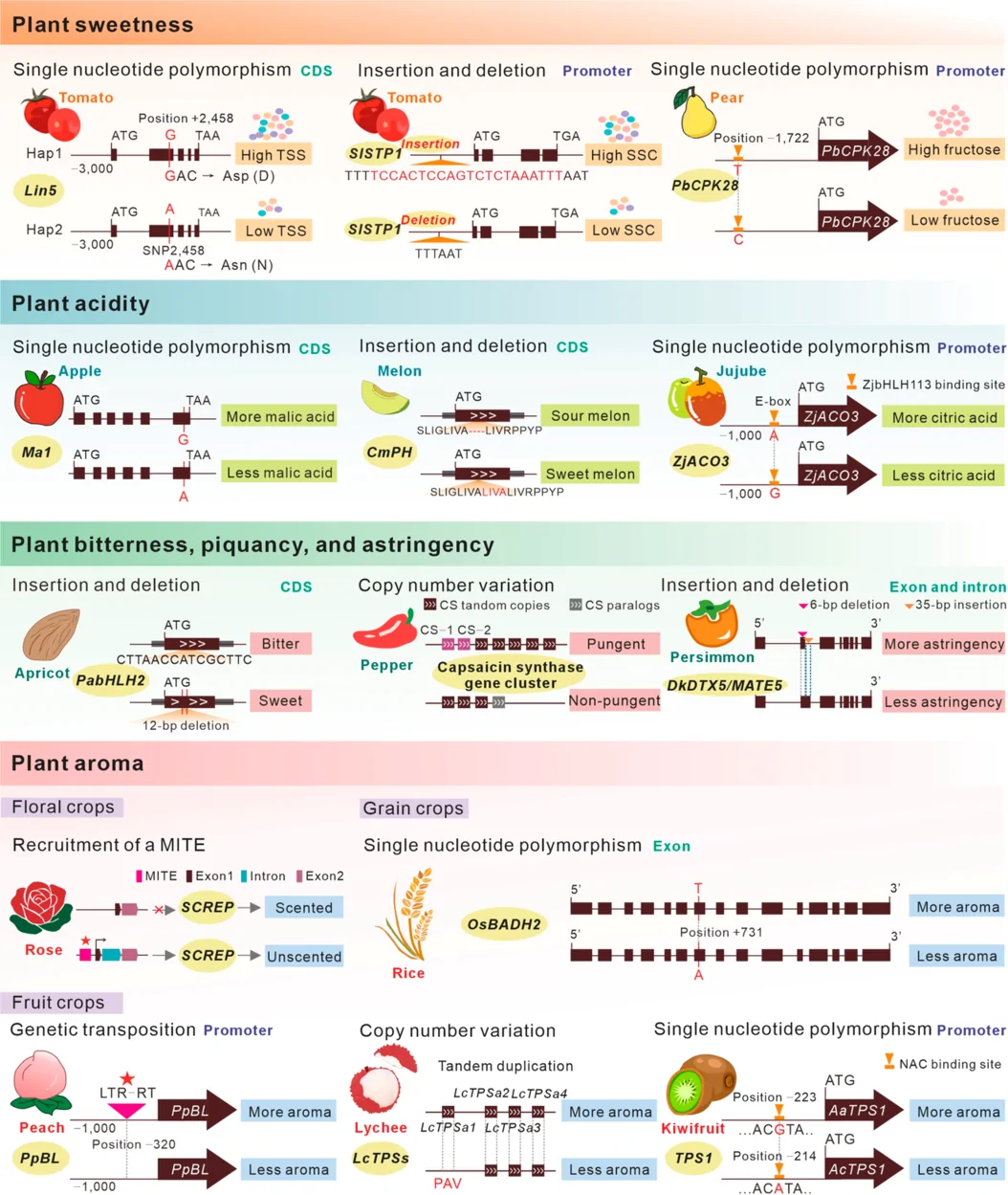
Specific examples of different genomic variations influencing plant flavor Abbreviations: *Aa*, *Actinidia arguta*; *Ac*, *Actinidia chinensis*; ACO, the cytoplasmic citrate degradation gene aconitase; BADH, betaine aldehyde dehydrogenase; bHLH, basic Helix‐Loop‐Helix; BL, an NAC transcription factor (Prupe.5G006200); CDS, coding sequence; *Cm*, *Cucumis melo*; CPK, calcium‐dependent protein kinase; CS, capsaicin synthase; *Dk*, *Diospyros kaki*; DTX, Detoxification efflux carrier; Hap, Haplotype; *Lc*, *Litchi chinensis*; *Lin5*, the tomato gene Solyc09g010080; LTR‐RT, long‐terminal repeat retrotransposon; Ma, aluminum‐activated malate transporter; MATE, multidrug and toxic compound extrusion; MITE, miniature inverted‐repeat transposable elements; *Os*, *Oryza sativa*; *Pa*, *Prunus armeniaca*; PAV, presence‐absence variation; *PH*, a pH‐associated gene; *Pp*, *Prunus persica*; SNP, single nucleotide polymorphism; TD, tandem duplication; TPS, terpene synthase; SCREP, a taxon‐restricted gene; *Sl*, *Solanum lycopersicum*; STP, sugar transporter protein; *Zj*, *Ziziphus jujuba*.

**Table 1:** List of different genomic variations playing an important role in plant sweetness

Species	Positions	Key genes	Types	Specific descriptions	References
Tomato	CDS	*Lin5*	SNP	The *Lin5* ^2458G^ haplotype exhibited significantly higher fruit TSS content than the *Lin5* ^2458A^ haplotype	(Wang et al., 2024)
Tomato	Promoter	*SlSTP1* ZAT10‐LIKE	InDel	Accessions with the 21‐bp insertion in *SlSTP1* have higher SSC than those with the 21‐bp deletion	(Wang et al., 2023d)
Tomato	Promoter	*SlCDPK26 SlCDPK27*	InDel	Accessions with Hap2 or Hap3 (12‐bp insertion) had significantly higher SSC than those with Hap1	(Zhang et al., 2024a)
Pear	Promoter	*PbCPK28 PbTST4 PbVHA‐A1*	SNP	SNP13T/C in the *PbCPK28* regulatory region led to fructose content diversity in pear	(Li et al., 2023a)
Apple	Promoter	*MdMYB109 MdWRKY33 MdSUT2.2*	SNP	SNPs in the promoter of *MdMYB109* influence its expression by attenuating MdWRKY33 binding, which upregulates the sucrose transporter *MdSUT2.2* and ultimately increases fruit sucrose content	(Zhang et al., 2025b)
Apple	Promoter	*MdMYB2 MdNADP‐ME*	SNP	The SNP alters MdMYB2 binding, thereby affecting *MdNADP‐ME* transcription and fructose levels at fruit ripening	(Fu et al., 2024)
Lychee	Intron	*LcSAI*	Deletion	Accessions with the deletion accumulated more sucrose and less reducing sugar	(Yan et al., 2025)
Watermelon		*CITST2*	CNV	The increased copy number contributes to the high sugar content in sweet watermelon varieties	(Deng et al., 2022)
*Stevia rebaudiana*		*CYPs and UGTs*	TD	Expansion and tandem duplication of *CYPs* and *UGTs* can assist in higher accumulations of SGs to meet growing industrial demand	(Masand et al., 2024)

Abbreviations: CDS, coding sequence; CDPK/CPK, calcium‐dependent protein kinase; CNV, copy number variation; CYP, cytochrome P450; InDel, insertion and deletion; *Lin5*, the tomato gene Solyc09g010080; *Md*, *Malus domestica*; MYB, v‐myb avian myeloblastosis viral oncogene homolog; NADP‐ME, NADP‐dependent malic enzyme; *PbVHA‐A1*, the vacuolar hydrogen proton pump; SAI, soluble acid invertase, a key enzyme for sucrose conversion into reducing sugars; SGs, steviol glycosides; SNP, single nucleotide polymorphism; SSC, soluble solids content; STP, sugar transporter protein; SUT, sucrose transporter; TD, tandem duplication; TSS, total soluble solids; TST, tonoplast sugar transporter; UGT, UDP‐glucuronosyltransferase; ZAT10‐LIKE, a zinc finger protein transcription factor (ZFP TF).

On the one hand, genomic variation in gene coding regions may alter protein products, thus influencing plant phenotypic traits (Tong et al., 2024). A study of 288 tomato accessions reported a progressive decrease in the total soluble solids (TSS) content of ripe fruits during domestication (Wang et al., 2024). Subsequently, a genome‐wide association study (GWAS) pinpointed a key genomic region on chromosome 9, harboring a natural variation (SNP2458) in the extracellular invertase gene *Lin5* (Solyc09g010080) that was highly associated with TSS levels. During domestication, a guanine (G) to adenine (A) mutation at this locus in wild tomatoes resulted in an aspartic acid (D) to asparagine (N) substitution at position 366 of the Lin5 protein. Based on this variation, tomato populations were categorized into two haplotype groups, and the *Lin5* ^2458G^ haplotype exhibited significantly higher fruit TSS content than the *Lin5* ^2458A^ haplotype (Figure 1; Table 1). Throughout the domestication process, the frequency of the *Lin5* ^2458G^ haplotype decreased while the *Lin5* ^2458A^ haplotype became predominant. This study further demonstrated that the natural variation SNP2458 in *Lin5* represents a major genetic locus responsible for reduced TSS content during tomato domestication (Wang et al., 2024), providing both a genetic target for improving fruit quality and technical support for gene‐editing approaches aimed at enhancing key agronomic traits in tomato.

On the other hand, genomic variation in non‐coding regions may alter the positioning or function of regulatory elements, thereby indirectly influencing gene expression (Zhao et al., 2025b). For instance, in tomato, a 21‐bp InDel in the promoter of the sugar transporter protein (*SlSTP1*) was found to significantly influence both its expression level and fruit soluble solids content (SSC) (Figure 1; Table 1; Wang et al., 2023d). In pear, Li et al. (2023a) have constructed a high‐density genetic linkage map and identified a major quantitative trait locus (QTL) controlling fruit SSC on linkage group LG15. Through integrated analysis of transcriptomic data from diverse pear varieties and phenotypic data of sugar content during fruit ripening, the calcium‐dependent protein kinase gene *PbCPK28* was identified as a key candidate gene regulating sugar accumulation. Notably, a single nucleotide polymorphism (SNP13 T/C) in the promoter region of *PbCPK28* was found to regulate its expression levels across different pear cultivars, consequently leading to natural variation in fructose accumulation in pear fruits (Figure 1; Table 1; Li et al., 2023a). In apple, a sophisticated transcriptional network regulating sucrose accumulation has been elucidated (Zhang et al., 2025b). In *Malus domestica*, three SNPs in the promoter of v‐myb avian myeloblastosis viral oncogene homolog 109 (*MdMYB109*) (SNP − 326 C/T, SNP − 705 A/G, and SNP − 706 G/T) collectively influence the binding of the repressive WRKY transcription factor 33 (MdWRKY33), resulting in enhanced *MdMYB109* expression. The upregulated MdMYB109 protein subsequently binds to and activates the promoter of the sucrose transporter protein (*MdSUT2.2*), thereby increasing fruit sucrose content (Table 1; Zhang et al., 2025b). Moreover, Fu et al. (2024) identified a major QTL on LG15 (marked by SNP np15_44) significantly contributing to fructose content and TSS through genome resequencing of the cultivated variety “Gala”, wild accession “Xiahongrou”, and their hybrid progenies. Further investigation revealed that a SNP (A/C) in the *Malus domestica* NADP‐dependent malic enzyme (*MdNADP‐ME*) promoter alters its binding affinity with transcription factor MdMYB2, consequently affecting *MdNADP‐ME* transcription and ultimately fructose levels during fruit maturation (Table 1; Fu et al., 2024). Based on these discoveries, the team developed a derived cleaved amplified polymorphic sequences (dCAPS) marker for selecting high‐sugar apple varieties (Fu et al., 2024), providing both theoretical foundations and practical tools for molecular breeding of fruit quality. More recently, the lychee soluble acid invertase (*SAI*) gene was identified as encoding a key enzyme involved in the conversion of sucrose into reducing sugars. Further characterization revealed a 524‐bp deletion in its second intron that is in strong linkage disequilibrium with the GWAS peak and predominantly found in high‐sucrose (SA‐type) accessions (Table 1; Yan et al., 2025). This deletion is associated with a significant increase in sucrose and a decline in reducing sugar content, defining the characteristic sugar accumulation phenotype (Yan et al., 2025). Furthermore, a PCR marker developed based on this deletion accurately discriminates lychee varieties with different sugar profiles, providing a valuable tool for marker‐assisted breeding aimed at optimizing sugar composition (Yan et al., 2025). These findings systematically reveal the molecular regulatory networks underlying sugar quality formation in fruits, significantly advancing our theoretical understanding of the genetic basis of flavor traits. The identification of key genes and their superior alleles regulating sugar metabolic pathways is progressively shifting fruit breeding from traditional empirical approaches toward precision molecular design, offering substantial potential for genetic improvement of crop quality.

Other major forms of structural variation include CNVs, PAVs, chromosomal inversions, translocations, and so on (Alkan et al., 2011). For example, researchers explored copy number variation at the *Citrullus lanatus* tonoplast sugar transporter (*ClTST2*) locus in watermelon. Their findings confirmed differential *ClTST2* copy numbers between sweet and non‐sweet germplasms, suggesting that increased copy number contributes to the high sugar content in sweet watermelon varieties (Table 1; Deng et al., 2022). Furthermore, *Stevia rebaudiana* Bertoni serves as an important source of plant‐derived low/no‐calorie natural sweeteners, collectively known as steviol glycosides (SGs) (Lemus‐Mondaca et al., 2012). Through evolutionary analysis and gene family characterization, studies have revealed that expansion and tandem duplication of cytochrome P450 (*CYP*) and UDP‐glucuronosyltransferase (*UGT*) genes correlate with enhanced accumulations of SGs (Table 1; Masand et al., 2024). These genetic insights provide valuable information for dissecting desirable traits and breeding superior genotypes with improved sweetening properties.

At the practical level of breeding, these fundamental research findings demonstrate broad application prospects. On the one hand, the identified key genes serve as direct targets for genetic improvement, enabling precise regulation through technologies such as CRISPR/Cas9‐mediated genome editing (Ahmar et al., 2023; Dellino et al., 2025). On the other hand, molecular markers significantly associated with sugar content and composition provide effective tools for marker‐assisted selection (Tiwari et al., 2022), substantially enhancing the breeding efficiency for new varieties characterized by high sugar content and well‐balanced flavor profiles. Future research needs to focus on integrated analysis of multi‐omics data to deeply elucidate the coordinated regulatory relationships between sugar metabolism and other important quality traits, such as acidity and aroma. Moreover, strengthening comparative genomic studies of allelic variations across different species will help reveal the evolutionary patterns of sugar accumulation mechanisms, thereby providing new insights for broadening the genetic foundation of plant flavor improvement.

## ADVANCES IN GENOMIC VARIATION AND PLANT ACIDITY

Organic acids serve as major components of plant flavor quality, working in concert with sugars to shape the sweet‐sour taste profile (Zhou et al., 2023). Meanwhile, they participate in essential physiological processes, including energy metabolism, ion homeostasis, and signal transduction, thereby influencing plant development, maturation, and postharvest characteristics (Tang et al., 2025). Domestication traits related to organic acid content have been extensively documented across fruit crops such as apple, tomato, and mandarin orange (Figure 1; Table 2). The profile of main organic acids shows significant variation across plant species. In Rosaceae fruits (e.g., apple, pear), malic acid is the predominant acid, imparting a fresh acidity (Li et al., 2020a). In citrus (e.g., orange, pomelo), citric acid is dominant, resulting in a milder taste (Violeta et al., 2010). In berries such as grape and kiwifruit, tartaric acid is the major component, delivering a sharp, tangy flavor (Mikulic‐Petkovsek et al., 2012; Walker and Famiani, 2018). The composition and concentration of organic acids represent classic quantitative traits governed by polygenic inheritance. In natural populations, QTL, GWAS, and evolutionary analyses have been widely employed to identify genetic loci and variations underlying organic acid content in plants.

**Table 2:** List of different genomic variations playing an important role in plant acidity

Species	Positions	Key genes	Types	Specific descriptions	References
Apple	CDS	*Ma1*	SNP	Most wild apple relatives possess the GG genotype and exhibit elevated malic acid content at maturity	(Ma et al., 2015)
Apple	Promoter	*MdSAUR37*	Insertion	A 36‐bp insertion in the promoter region significantly enhanced the expression of *MdSAUR37*, resulting in increased fruit acidity	(Jia et al., 2018)
Apple	Promoter	*MdMYB44 Ma1*	SNP	SNP G and SNP‐ were associated with higher malate content, whereas SNP A and SNP T were associated with lower malate content	(Jia et al., 2021)
Apple	Promoter	*MdMYB21 MdMa1*	InDel	A 2‐bp variation in the *MdMYB21* promoter region altered the expression of its target gene, *MdMa1*	(Peng et al., 2023)
Apple	Promoter	*MdbHLH74 MdMa7*	InDel	An insertion in the promoter of a malate dehydrogenase gene regulates malic acid content	(Gao et al., 2024)
Apple	Promoter	*MdMYB2 MdNADP‐ME*	SNP	An A/C SNP in the promoter region (MEp‐799) is associated with malate level	(Fu et al., 2024)
Tomato	Promoter	*SlWRKY42 S1ALMT9*	InDel	The 3‐bp Indel affected the expression of *SlALMT9* and consequently influenced malate accumulation	(Ye et al., 2017)
Jujube	Promoter	*ZjWRKY7 ZjALMT4*	SNP	A stable linked‐genetic variation at position − 1,054 bp (G/C/T) associated with fruit malate accumulation	(Zhang et al., 2023)
Jujube	Promoter	*ZjbHLH113 ZjACO3*	SNP	‘A’ represented a high‐citrate genotype and ‘G’ represented a low‐citrate genotype	(Liu et al., 2024a)
Sweet melon	CDS	*CmPH*	InDel	A four amino‐acid duplication in CmPH distinguishes between primitive acidic varieties and modern dessert melons	(Cohen et al., 2014)
Peach	Exon	*PpTST1*	SNP	The G1584T transversion in the third exon of *PpTST1*, resulting in a Q528H substitution, is associated with low organic acid content in peach	(Wang et al., 2022)
Pear		*Ma1*	PAV	The *Ma1* allele was found to be absent in varieties with low organic acid content	(Li et al., 2024a)

Abbreviations: ACO, the cytoplasmic citrate degradation gene aconitase; ALMT, aluminum‐activated malate transporter; bHLH, basic Helix‐Loop‐Helix; CDS, coding sequence; *Cm*, *Cucumis melo*; InDel, insertions and deletion; Ma, aluminum‐activated malate transporter; *Md*, *Malus domestica*; MYB, v‐myb avian myeloblastosis viral oncogene homolog; PAV, presence‐absence variation; *PH*, a pH‐associated gene; *Pp*, *Prunus persica*; *Sl*, *Solanum lycopersicum*; SNP, single nucleotide polymorphism; TST, tonoplast sugar transporter; *Zj*, *Ziziphus jujuba*.

Malate represents a key determinant of flavor and processing quality in apple fruits, making the precise regulation of its concentration an important breeding objective (Jia et al., 2021). Researchers have identified the aluminum‐activated malate transporter *Ma1* (*Al‐ACTIVATED MALATE TRANSPORTER 9*) as a candidate gene controlling fruit acidity in apple. As shown in Figure 1, a critical SNP occurred at the coding sequence of the *Ma1* gene (Ma et al., 2015). When the SNP (G/A) carried a G base, the resulting protein localizes to the tonoplast and transports malic acid into the vacuole, in turn promoting fruit acid accumulation. In contrast, an A base at this SNP (G/A) introduces a premature stop codon (producing a truncated protein designated *ma1*), which disrupts vacuolar targeting and impairs malate transport into vacuoles, leading to reduced acidity. However, the observation that some *ma1ma1* genotypes still maintain high fruit acidity indicates that *Ma1* is not the sole determinant of malate levels (Figure 1; Table 2). Subsequently, Jia et al. (2021) revealed that MdMYB44, an R2R3‐MYB transcription factor, negatively regulates malate accumulation by repressing the promoter activity of malate‐associated genes, including *Ma1*, *Ma10* (*P‐type ATPase 10*), *MdVHA‐A3* (*V‐type ATPase A3*), and *MdVHA‐D2* (*V‐type ATPase D2*). Genotypic analysis of diverse apple germplasm identified two SNPs in the *MdMYB44* promoter (T/− and A/G), forming distinct haplotypes that influence malate content. The haplotype combining SNP (T) and SNP (A) shows high *MdMYB44* expression and low malate accumulation, whereas the haplotype with SNP (−) and SNP (G) exhibits low *MdMYB44* expression and high malate levels (Table 2). Collectively, this research elucidated the regulatory role of MdMYB44 in malate accumulation, characterized the functional impact of promoter variations on its transcriptional activity, and identified transcription factors binding to the element containing SNP (T). These findings provided important insights into the molecular network controlling malate accumulation in apple and established a foundation for developing efficient molecular markers targeting acidity traits.

Similarly, Ye et al. (2017) identified *TFM6* (*tomato fruit malate 6*) as a major locus regulating malate accumulation in tomato. This gene encodes a *Solanum lycopersicum* aluminum‐activated malate transporter, SlALMT9, localized to the cell membrane. A 3‐bp InDel within the promoter of *SlALMT9* is situated precisely within a W‐box cis‐element that is directly bound by the transcription factor SlWRKY42. This binding reduced the expression of *SlALMT9* and consequently reduced malate accumulation in tomato fruits (Table 2; Ye et al., 2017). This study highlighted *SlALMT9* as a key target of selection during tomato domestication and improvement, providing a mechanistic basis for flavor enhancement. As the most economically important fruit crop in the Rhamnaceae family, the jujube (*Ziziphus jujuba* Mill.) derives from wild jujube through domestication (Ji et al., 2017). During this process, organic acid content significantly decreased, making the organic acid accumulation mechanism a critical factor determining fruit sweetness and acidity (Liu et al., 2024a). A key *E*‐box mutation (−484 A > G) was identified in the promoter of the aconitase gene *ZjACO3*, where the wild genotype CAAGTG shifts to CAGGTG in cultivated varieties (Liu et al., 2024a). This mutation shows a strong association with citrate content, with the ‘A’ allele corresponding to high‐citrate and ‘G’ to low‐citrate genotypes (Figure 1; Table 2). Based on this variation, Liu et al. (2024a) developed and validated a Kompetitive allele‐specific PCR (KASP) marker targeting the *ACO* locus, providing an efficient tool for rapid selection of low‐acidity jujube cultivars. This genetic insight bridged fundamental discovery and practical application in plant quality breeding.

## ADVANCES IN GENOMIC VARIATION AND PLANT BITTERNESS, PIQUANCY, AND ASTRINGENCY

The rapid development of functional genomics is driving plant flavor research toward deeper molecular‐level analysis of complex traits such as bitterness, pungency, and astringency (Table 3). In Cucurbitaceae crops, cucurbitacin C (CuC) serves as the primary bitter compound, particularly abundant in leaves and fruits of wild cucumber varieties (Chai and Sun, 2024). In contrast, most cultivated cucumbers produce non‐bitter fruits. Through genomic analysis of 115 cucumber accessions combined with metabolomic and enzymatic studies, Shang et al. (2014) have elucidated a conserved cucurbitacin biosynthetic pathway involving at least nine key genes. The *Bi* gene initiates this pathway by encoding a cyclase responsible for producing the basic triterpenoid skeleton. Subsequently, multiple *P450* genes and an acetyltransferase (*ACT*) gene progressively modify this skeleton, ultimately yielding the bitter compound cucurbitacin C. In modern cultivated cucumbers, structural mutations or deletions in the *Bt* gene promoter prevent activation of downstream biosynthetic genes, effectively eliminating cucurbitacin production and resulting in non‐bitter fruits (Shang et al., 2014). Similarly, cucurbitacin C represents the main bitter principle in melon. Using forward genetics approaches, researchers identified a genomic region controlling melon bitterness and discovered a PAV associated with the bitter phenotype. The 62‐kb deleted region contains nine consecutive genes, including five cytochrome *P450* genes, providing new theoretical support and molecular targets for breeding non‐bitter melon varieties (Li et al., 2020b). Beyond cucurbit crops, the bitter trait in apricot is driven by a 15‐bp Indel in the bHLH domain of the *PabHLH2* gene (Figure 1; Zhang et al., 2025a). The inserted *PabHLH2a* allele acts as a homodimeric transcription factor that activates the amygdalin pathway by binding to the CATGTG cis‐element in the promoters of *PaCYP71 AN24* and *PaCYP79D16*, thereby upregulating these critical genes and promoting the accumulation of their precursor and product, ultimately conferring the bitter taste (Zhang et al., 2025a). The molecular markers developed from this discovery enable early selection of kernel traits, providing theoretical and technical support for molecular breeding of apricot varieties with either low amygdalin content (for food use) or high amygdalin content (for pharmaceutical applications).

**Table 3:** List of different genomic variations playing an important role in plant bitterness, piquancy, and astringency

Species	Positions	Key genes	Types	Specific descriptions	References
Cucumber	Promoter	*Bt*	Deletion	In cultivated cucumbers, mutations or deletions in the *Bt* gene promoter disrupt activation of downstream genes, resulting in non‐bitter fruits	(Shang et al., 2014)
Melon		*CYPs*	PAV	A bitterness‐controlling genomic region with a PAV linked to the bitter phenotype	(Li et al., 2020b)
Apricot	CDS	*PabHLH2*	Insertion	15‐bp insertion positively regulates both *PaCYP71AN24*/*PaCYP79D16* expression and prunasin accumulation	(Zhang et al., 2025a)
Pepper		*CS*	TD	Tandem duplication of the *CS* gene enhances pungency in pepper.	(Chen et al., 2024)
Pepper		*Pun1*	SV	The absence of pungency in ‘Sampo Oamanaga’ peppers associates with this *pun1* ^*5*^ allele	(Yamaguchi et al., 2024)
Persimmon	Exon and intron	*DkDTX5/MATE5*	InDel	Natural allelic variation drives differential proanthocyanidin accumulation between A‐type and NA‐type fruit	(Liu et al., 2023)

Abbreviations: *A*‐type, normal astringent; *Bt*, bitter gene; bHLH, basic Helix‐Loop‐Helix; CDS, coding sequence; CS, capsaicin synthase; CYP, cytochrome P450; *Dk*, *Diospyros kaki*; DTX, Detoxification efflux carrier; InDel, insertion and deletion; MATE, multidrug and toxic compound extrusion; NA‐type, non‐astringent; *Pa*, *Prunus armeniaca*; PAV, presence‐absence variation; Pun, Pungency; SV, structural variation; TD, tandem duplication.

Capsaicin is the compound responsible for the characteristic pungency of chili peppers, and its presence determines whether peppers are used as spices or vegetables. Capsaicin synthase (CS), the key enzyme in capsaicin biosynthesis (Aza‐González et al., 2011), exists uniquely in Solanaceous crops as tandem repeats. Notably, domesticated annual peppers contain seven tandem copies of the *CS* gene, while wild peppers possess only four copies, suggesting that copy number variation contributes to the pungency divergence between domesticated and wild peppers (Chen et al., 2024). Transcriptomic analysis revealed high expression of the *CS* gene in pungent pepper fruits, whereas non‐pungent plants such as tomato, potato, physalis, and wild peppers show minimal expression. Sequence alignment demonstrated that functional *CS* alleles (*CS‐1/CS‐2*) in pungent peppers maintain conserved coding sequences and regulatory regions, whereas structural variations in both coding and regulatory regions are observed in non‐pungent peppers and related Solanaceous species (Figure 1; Table 3; Chen et al., 2024). Furthermore, Japanese researchers identified an 18.5‐kbp deletion on chromosome 2 containing a novel allele, *Pun1* ^*5*^ (Yamaguchi et al., 2024). Then, they confirmed that the absence of pungency in “*Sampo Oamanaga*” peppers associates with this *pun1* ^*5*^ allele. This study represented the first discovery of a large genomic deletion encompassing a gene within a loss‐of‐function *pun1* allele, providing new insights into the regulatory mechanisms of capsaicin biosynthesis in chili peppers.

The astringency in persimmon is primarily caused by the accumulation of proanthocyanidins (PAs) in the vacuole, which markedly differs between normal astringent (A‐type) and its non‐astringent (NA‐type) mutant (Liu et al., 2023). After cloning the *DkDTX5/MATE5* gene from A‐type fruit, researchers identified two transcripts (*DkDTX5/MATE5‐1* ^*A*^ and *DkDTX5/MATE5‐2* ^*A*^). As shown in Figure 1, sequence analysis revealed that both variants contain eight exons, but *DkDTX5/MATE5‐2* ^*A*^ harbors a 6‐bp deletion in the second exon and a 35‐bp insertion in the second intron. *DkDTX5/MATE5‐2* ^*A*^ was expressed more highly than *DkDTX5/MATE5‐1* ^*A*^, establishing it as the predominant allele in A‐type fruit. Subsequent transient overexpression assays demonstrated that DkDTX5/MATE5‐1^A^, but not DkDTX5/MATE5‐2^A^, could transport proanthocyanidin precursors (Liu et al., 2023). This indicated that natural allelic variation in *DkDTX5/MATE5* regulated the differential accumulation of proanthocyanidins between A‐type and NA‐type fruit (Table 3). This study elucidated a key molecular mechanism underlying persimmon astringency and provided a theoretical foundation and genetic resource for future allele marker‐assisted breeding of non‐astringent persimmons.

The formation of key plant traits such as bitterness, pungency, and astringency is not governed solely by individual genes, but is determined by complex regulatory networks involving multiple genomic variations. These findings provide a critical theoretical foundation and precision molecular tools for plant genetic improvement. By systematically elucidating key genetic loci and their regulatory mechanisms, breeders can now more precisely balance the relationship between flavor quality and essential agronomic traits such as stress resistance and yield. Therefore, these research advances not only enable the development of new cultivars with superior taste and broad environmental adaptability but also mark a transition in plant breeding practice from traditional phenotype‐dependent selection toward a genomics‐informed precision design paradigm.

## ADVANCES IN GENOMIC VARIATION AND PLANT AROMA

VOCs represent essential components of plant flavors, defining distinctive aromas and influencing the consumer's sensory experience, purchasing decisions, and market acceptance (Gang, 2005; Duan et al., 2023). In ornamental plants, rose stands as one of the most historically significant cultivated flowers worldwide, valued not only for landscaping but also for its substantial economic importance in the cosmetics and fragrance industries. However, a notable industry challenge is that over 35,000 modern rose cultivars have largely lost the intense fragrance of their wild ancestors during hybridization breeding (Li et al., 2025b). Integrating genomic assembly, evolutionary analysis, and functional validation, Li et al. (2025b) constructed an evolutionary‐functional model elucidating the diversification of rose aroma. Their findings revealed that the *SCREP* gene (a taxon‐restricted gene), specific to the rose lineage, acts as a transcription factor regulating key enzymes in the phenylpropanoid pathway. The presence or absence of *SCREP*, combined with insertions or deletions of miniature inverted‐repeat transposable elements (MITEs), collectively determines variation in eugenol content among Rosa species, eventually driving the diversification of rose fragrance profiles (Figure 1; Table 4). Similarly, jasmine enjoys global acclaim for its unique aroma and ornamental value (Fang et al., 2025). Studies have identified *Jasminum sambac TPS3* (*JsTPS3*) and *JsTPS17* as specifically and highly expressed in jasmine flowers, responsible for synthesizing major terpene volatiles such as α‐farnesene and linalool (Fan et al., 2025). Gene duplication events (such as the tandem duplication of *JsTPS1* and *JsTPS2*) may further enhance the diversity of jasmine aroma in a dosage‐dependent manner. In addition, the *benzyl alcohol O‐acetyltransferase* (*BEAT*) gene, which contributes to the biosynthesis of characteristic jasmine aromatic compounds, is also highly expressed in floral organs. Notably, the single‐petal jasmine (SJ) variety contains two *BEAT* copies, whereas other varieties possess only one, which may explain the superior fragrance of single‐petal jasmine (Table 4; Fan et al., 2025). These findings systematically illustrate how genomic variation shapes the diversity of floral aromas, providing a scientific basis for molecular breeding of aroma traits.

**Table 4:** List of different genomic variations playing an important role in plant aroma

Species	Positions	Key genes	Types	Specific descriptions	References
Rose		*SCREP*	PAV InDel	The PAV of *SCREP*, combined with insertions or deletions of MITE transposons, collectively determines variation in eugenol content among Rosa species, ultimately driving the diversification of rose aroma profiles	(Li et al., 2025b)
Rose		*NUDX1‐1a*	CNV	The copy number of *NUDX1‐1a* correlates with citronellol content across rose species	(Shang et al., 2024)
Jasmine		*JsTPS1* and *JsTPS2*	TD	Gene duplication events may further enhance the diversity of jasmine aroma through a dosage effect	(Fan et al., 2025)
Jasmine		*BEAT*	CNV	The SJ variety contains two *BEAT* copies, whereas other varieties possess only one, which may explain the superior fragrance of SJ	(Fan et al., 2025)
*Freesia* species	CDS	*TPS6*	SNP	The mutant FspTPS6‐I287A produced a similar product profile to FveTPS6, indicating that the I287A single‐point mutation significantly altered the floral volatile terpene composition of FspTPS6	(Bao et al., 2023b)
Kiwifruit	Promoter	*AaTPS1*	SNP	This SNP influenced the binding affinity of NAC transcription factors to the promoter, thereby promoting monoterpene accumulation	(Nieuwenhuizen et al., 2015)
Peach		*PpBL*	LTR‐RT	An upstream LTR‐RT drives high *PpBL* expression, thereby shaping both flesh color and aroma	(Cao et al., 2024)
Durian		*MGL*	CNV	The expansion of gene families in sulfur‐related pathways, notably through the *MGL* gene, is associated with the enhanced odor profile of durian	(Teh et al., 2017)
Lychee		*LcTPSa1‐4*	PAV CNV	PAV and CNV of *LcTPSa1* influence the expression of the entire *TPS* gene cluster, thereby affecting sesquiterpene biosynthesis	(Hu et al., 2025)
Strawberry		*FaFAD1*	PAV	PAV of *FaFAD1* influenced the content of γ‐decalactone	(Oh et al., 2021)
Cucumber	Intron	*CsLOX08*	Insertion	An 8‐bp insertion may influence the content of (*E*,*Z*)‐2,6‐nonadien‐1‐ol	(Sun et al., 2022)
Pumpkin		*CmoBADH*	Insertion	The structural variation (4,591‐bp insertion) disrupted normal gene transcription, resulting in loss‐of‐function of the encoded BADH enzyme, consequently promoting 2‐AP accumulation	(Zhao et al., 2025a)
Rice	Exon	*OsBADH2*	SNP	An A‐T substitution of *OsBADH2*, resulting in a leucine‐to‐lysine substitution that enhances 2‐AP biosynthesis	(Li et al., 2024c)

Abbreviations: 2‐AP, 2‐acetyl‐1‐pyrroline; *Aa*, *Actinidia arguta*; BADH, betaine aldehyde dehydrogenase; BEAT, benzyl alcohol O‐acetyltransferase; BL, an NAC transcription factor (Prupe.5G006200); CDS, coding sequence; CNV, copy number variation; *Cmo*, *Cucurbita moschata*; *Cs*, *Cucumber sativus*; *Fa*, *Fragaria × ananassa*; FAD, FATTY ACID DESATURASE GENE 1; InDel, insertions and deletion; *Js*, *Jasminum sambac*; *Lc*, *Litchi chinensis*; LOX, lipoxygenase; LTR‐RT, long‐terminal repeat retrotransposon; MGL, methionine γ‐lyase; MITE, miniature inverted‐repeat transposable elements; NUDX, nudix hydrolase; *Os*, *Oryza sativa*; PAV, presence‐absence variation; *Pp*, *Prunus persica*; SCREP, a taxon‐restricted gene; SJ, single‐petal jasmine; SNP, single nucleotide polymorphism; TD, tandem duplication; TPS, terpene synthase.

In fruit crops, various types of genomic variation significantly influence the aroma profiles (Figure 1; Table 4). Previous study revealed that differences in ripe fruit aroma between *Actinidia arguta* and *Actinidia chinensis* primarily originated from a natural SNP variation in the *AaTPS1* promoter (Nieuwenhuizen et al., 2015). This variation enhanced the binding affinity of NAM/ATAF1/2/CUC (NAC) transcription factors to the promoter, thereby promoting monoterpene accumulation in ripe *A. arguta* fruits and subsequently shaping their aromatic characteristics (Figure 1; Table 4; Nieuwenhuizen et al., 2015). This finding not only offers a fresh perspective on the genetic basis of fruit aroma but also provides a theoretical foundation for flavor improvement in kiwifruit and other fruit tree species. In recent years, an increasing number of studies have reported diverse genomic variations influencing fruit aroma across different species. In peach, a long‐terminal repeat retrotransposon (LTR‐RT) located 320‐bp upstream of the NAC transcription factor (*PpBL*) transcription start site was specifically present in red‐fleshed fruits but absent in white‐fleshed ones, leading to high expression of *PpBL* that influences both flesh color and aroma (Figure 1; Table 4; Cao et al., 2024). In durian, the combination of upregulated sulfur metabolism genes and multiple biosynthetic gene copies leads to enhanced volatile sulfur compound production (Table 4; Teh et al., 2017). This high level of volatile sulfur generated the characteristic aroma in ripe fruit. In lychee, PAV and CNV of *LcTPSa1* influence the expression of the entire *TPS* gene cluster, thereby affecting sesquiterpene biosynthesis and aroma formation (Figure 1; Table 4; Hu et al., 2025). In strawberry, PAV of *FaFAD1* influenced the content of γ‐decalactone, which is a key contributor to fruity aroma (Table 4). The above result suggested that allelic variation may offer a potential strategy for flavor improvement (Oh et al., 2021). In summary, genomic variation plays an indispensable role in determining the diversity of plant flavor. These findings shed new light on understanding the genetic underpinnings of plant flavor evolution and provide a rich repository of gene targets and genetic resources for future flavor‐oriented breeding programs.

In vegetable crops, cucumber (*Cucumis sativus*) fruits possess a distinct fresh aroma that constitutes a key component of their flavor quality. More than 70 VOCs have been identified in cucumber fruits, including aldehydes, alcohols, ketones, terpenes, esters, and furans (Ji et al., 2025). In particular, C6 aldehydes (imparting grass odor) and C9 aldehydes (contributing floral aromas) represent the predominant components. Using a recombinant inbred line population derived from Q16 × Q24 and resequencing data, Sun et al. (2022) constructed a genetic map comprising 1,301 SNP markers and detected 28 QTLs associated with major aldehyde volatiles across two growing seasons. For C9 aldehydes, a major QTL (qol8‐2.1) was identified for the characteristic aroma compound (*E*,*Z*)‐2,6‐nonadien‐1‐ol. Genomic scanning revealed nine lipoxygenase genes within this locus. Among them, the expression of one lipoxygenase (*CsLOX08*) showed a significant positive correlation with the content of (*E*,*Z*)‐2,6‐nonadien‐1‐ol. Furthermore, an 8‐bp insertion was identified in the fifth intron of the Q24 genome compared to Q16 (Sun et al., 2022). Based on this insertion variation in *CsLOX08*, the researchers developed a functional InDel marker, laying a theoretical foundation for understanding the synthesis and regulation of cucumber fruit aroma (Table 4). Likewise, the ‘taro‐like’ aroma in pumpkin represents another unique flavor profile highly valued by consumers, primarily attributed to the key volatile compound 2‐acetyl‐1‐pyrroline (2‐AP). Zhao et al. (2025a) constructed an F2 population from a cross between pumpkin accessions NO.44 (with taro‐like aroma) and NO.45 (without taro‐like aroma). Integrated genomic assembly and functional analysis revealed a 4,591 bp insertion in candidate gene CmoCh10G001620 (*CmoBADH*) in the aromatic pumpkin NO.44. This structural variation disrupted normal gene transcription, resulting in loss‐of‐function of the encoded betaine aldehyde dehydrogenase (BADH) enzyme, consequently promoting 2‐AP accumulation and ultimately generating the characteristic taro‐like aroma (Table 4; Zhao et al., 2025a). According to this key variation, a functional marker applicable for marker‐assisted selection was developed. This marker enables rapid and accurate selection of taro‐aroma individuals at the seedling stage, establishing a paradigm for flavor improvement and quality breeding in vegetable crops.

In grain crops, aroma represents a key factor determining the cooking quality and commercial value of rice. 2‐AP is the principal component of rice fragrance, and its distinctive popcorn‐like aroma is regarded as the key source of the characteristic scent in aromatic rice (Imran et al., 2023). Studies have identified an A‐T substitution at position 731‐bp in exon 7 of LOC_Os08g32870 (*OsBADH2*), resulting in a leucine‐to‐lysine substitution that alters the aldehyde dehydrogenase domain and enhances 2‐AP biosynthesis (Table 4; Li et al., 2024c). Beyond *OsBADH2*, SNP vg0528473372 (A‐T) also showed a significant association with 2‐AP content. This A/T substitution occurred within a *MITE*, where it modulated mRNA levels of *OsWRKY19*, subsequently regulating *OsBADH2* expression and ultimately influencing 2‐AP accumulation (Figure 1; Table 4; Li et al., 2024c). These findings provided novel molecular targets for improving rice fragrance. While previously reported *BADH2* mutations predominantly localize to exonic regions, recent work has identified and characterized *badh2‐I11*, a novel allelic variant of the *BADH2* gene. This allele features a unique 55‐bp deletion in intron 11 that induces alternative splicing during transcription, generating both partially normal transcripts and abnormal transcripts containing premature termination codons (Liang et al., 2025). This mechanism downregulated the expression of *Badh2* without complete loss of function, resulting in an intermediate ‘mild’ fragrance phenotype between common rice and traditional aromatic varieties (Liang et al., 2025). This discovery revealed a novel regulatory mechanism where intronic mutations modulate rice fragrance through alternative splicing, providing valuable genetic resources and molecular tools for breeding intermediate‐fragrance rice varieties.

In medicinal plants, genomic variations also play a pivotal role in shaping the accumulation of VOCs, thereby influencing flavor profiles that may possess pharmacological properties. For instance, in dill (*Anethum graveolens*), a medicinal and aromatic plant renowned for its high VOCs content and extensive use in essential oil extraction and pharmaceutical applications, Wang et al. (2025) revealed that the expansion and expression of *TPS* gene family members are closely associated with VOCs accumulation. Similarly, in tea (*Camellia sinensis*), Tao et al. (2025) demonstrated that insertions in the promoter of key genes are linked to the biosynthesis of VOCs, potentially affecting tea aroma. These findings highlight how structural variations in regulatory and coding regions can regulate secondary metabolism in medicinal plants, offering insights into the genetic basis of flavor traits that may also contribute to their therapeutic efficacy. Therefore, in‐depth research on genomic variations enables the precise regulation of plant secondary metabolic pathways. This approach allows for the upregulation of specific genes to enhance the biosynthesis of desirable aroma compounds, such as terpenes, esters, and phenylpropanoids, in flowers, fruits, and herbal vegetables. Conversely, undesirable flavor compounds in plants can be effectively reduced through gene silencing or by employing precision genome editing tools such as CRISPR‐Cas9 for targeted gene knockout.

## AI‐DRIVEN DESIGN AND PRECISION GENOME EDITING FOR FLAVOR ENHANCEMENT IN PLANTS

Artificial intelligence (AI) approaches have shown considerable promise in predicting plant flavor traits, although they have been applied at different biological levels. At the metabolite and sensory level, Ferrão et al. (2022) used random forest models to identify terpenoid compounds, including myrcenol, p‐xylene, and D‐limonene, as key contributors to blueberry aroma profiles. Similarly, Colantonio et al. (2022) evaluated 18 statistical and machine learning methods to predict sensory characteristics from metabolite data, with models such as XGBoost achieving prediction accuracies of 0.62–0.87 for sweetness, flavor intensity, and acidity. A recent survey further summarized machine learning and deep learning approaches for flavor prediction across multiple modalities and emphasized the importance of integrating multi‐omics and receptor‐ligand information to improve model interpretability and mechanistic insight (Osinomumu et al., 2025). At the genomic level, predictive models have also been developed for flavor traits. In blueberry, Ferrão et al. (2020) showed that genomic selection models, including those incorporating GWAS‐significant loci, together with marker‐assisted selection, can accurately predict flavor‐associated volatile organic compounds. In sugarcane, machine learning methods, including deep neural networks and random forests, were similarly applied to genomic prediction, yielding accuracies for total cane harvested and commercial cane sugar comparable to those of the genomic best linear unbiased prediction model (Inamori et al., 2024). Taken together, these studies show that AI can support flavor research both by predicting sensory traits from metabolite‐level information and by improving genome‐based prediction for breeding applications.

Significantly, Moore et al. (2019) used machine learning in combination with genomic data to identify genes associated with specialized metabolism in *Arabidopsis thaliana*. The resulting predictive model accurately recovered 87% of known specialized metabolism genes and further predicted 1,220 genes of previously unknown function. In this context, artificial intelligence‐based analysis of how genomic variation, including single nucleotide polymorphisms and structural variants, affects genes involved in specialized metabolism could help clarify their contributions to flavor formation and facilitate the identification of candidate targets for flavor enhancement. Recent methodological advances have further expanded the scope of AI applications in plant metabolism research. For example, EnzySeek, a large language model‐driven agent for enzyme catalysis, integrates a curated literature knowledge base with tool‐linked workflows spanning structure prediction, docking, molecular dynamics, and molecular mechanics calculations (Kang et al., 2026). By autonomously retrieving prior evidence, coordinating computational analyses, and interpreting the resulting outputs, this framework provides a promising strategy for investigating enzymes involved in flavor compound biosynthesis. In addition, Bao et al. (2023a) developed a transfer learning approach based on a hybrid Graph Transformer and Convolutional Neural Network that integrates molecular graph structures with SMILES. Pre‐trained on KEGG and fine‐tuned on plant datasets, the model enables accurate prediction of plant secondary metabolic pathways and natural product classes, and is accompanied by a user‐friendly tool for pathway prediction from compound SMILES. Together, these studies suggest that building more comprehensive and biologically informed AI frameworks will be an important direction for future flavor prediction and breeding. Beyond plant systems, AI tools in mammalian systems have also made substantial progress and provide useful methodological references for plant research. By combining lentiviral‐mediated multiplexed reporter assays with deep learning, Deng et al. (2024) profiled more than 100,000 candidate regulatory sequences and variants in human cortical cells and cerebral organoids, identified thousands of active enhancers, detected disease‐associated allelic effects, and trained a sequence‐to‐activity model with high predictive accuracy. Similarly, Agarwal et al. (2025) used large‐scale reporter assays to examine hundreds of thousands of candidate cis‐regulatory elements, revealing promoter strand‐orientation biases, broadly active core elements, and determinants of tissue specificity, while also showing that sequence‐based models can accurately predict cis‐regulatory function and variant effects. At the genome scale, AlphaGenome analyzes 1 Mb of DNA sequence to predict thousands of functional tracks at single‐base resolution across modalities and achieves state‐of‐the‐art performance in variant‐effect prediction (Avsec et al., 2026). Collectively, these studies show how high‐throughput functional assays combined with powerful sequence models can improve the interpretation of regulatory variation. Although most of these tools were developed in mammalian systems, they offer a useful roadmap for future plant studies aimed at decoding regulatory grammar and interpreting genomic variants that influence flavor diversity.

How can genomic variation and advanced technologies enable more precise optimization of plant flavor? More and more studies demonstrate that gene‐editing tools such as CRISPR‐Cas9 enable targeted modification of flavor‐related gene sequences with text‐editing‐like precision (Figure 2; Xue et al., 2025b). For instance, knockout of *SlMYB12* via CRISPR‐Cas9 successfully generated a pink‐fruit mutant. Metabolomic profiling of the mutant revealed a substantial accumulation of polyamines, polyphenols, and solanine, which are key compounds influencing tomato flavor (Figure 2; Zhu et al., 2018). Furthermore, Zhang et al. (2024a) used genome‐wide association analysis to identify *SlCDPK27* as a negative regulator of sugar accumulation in tomato fruit. Their work showed that knocking out *SlCDPK27* using CRISPR‐Cas9 significantly increased glucose and fructose content while maintaining fruit weight, thereby overcoming the typical trade‐off between sugar levels and fruit size (Figure 2; Zhang et al., 2024a; Li et al., 2025c). This strategy offered a new molecular breeding approach to enhance tomato sweetness without compromising yield. In kiwifruit, citrate serves as the primary contributor to organic acid content in ripe fruit. As shown in Figure 2, Fu et al. (2023) demonstrated that the transcription factor AcNAC1 participates in citrate accumulation by activating the expression of *AcALMT1*, which encodes a citrate transporter. By targeting AcNAC1 with CRISPR/Cas9‐mediated mutagenesis, the researchers successfully generated low‐citrate kiwifruit lines. For aroma improvement, Huang et al. (2025) knocked out *LOX* genes in kiwifruit, resulting in fruits with perceptibly reduced grass‐odor volatiles, which is a difference readily detectable by consumers (Figure 2). Together, these advances highlighted the potential of gene editing to reshape plant flavor profiles, with promising implications for global plant production and the enhancement of flavor traits in other plants.

**Figure 2:**
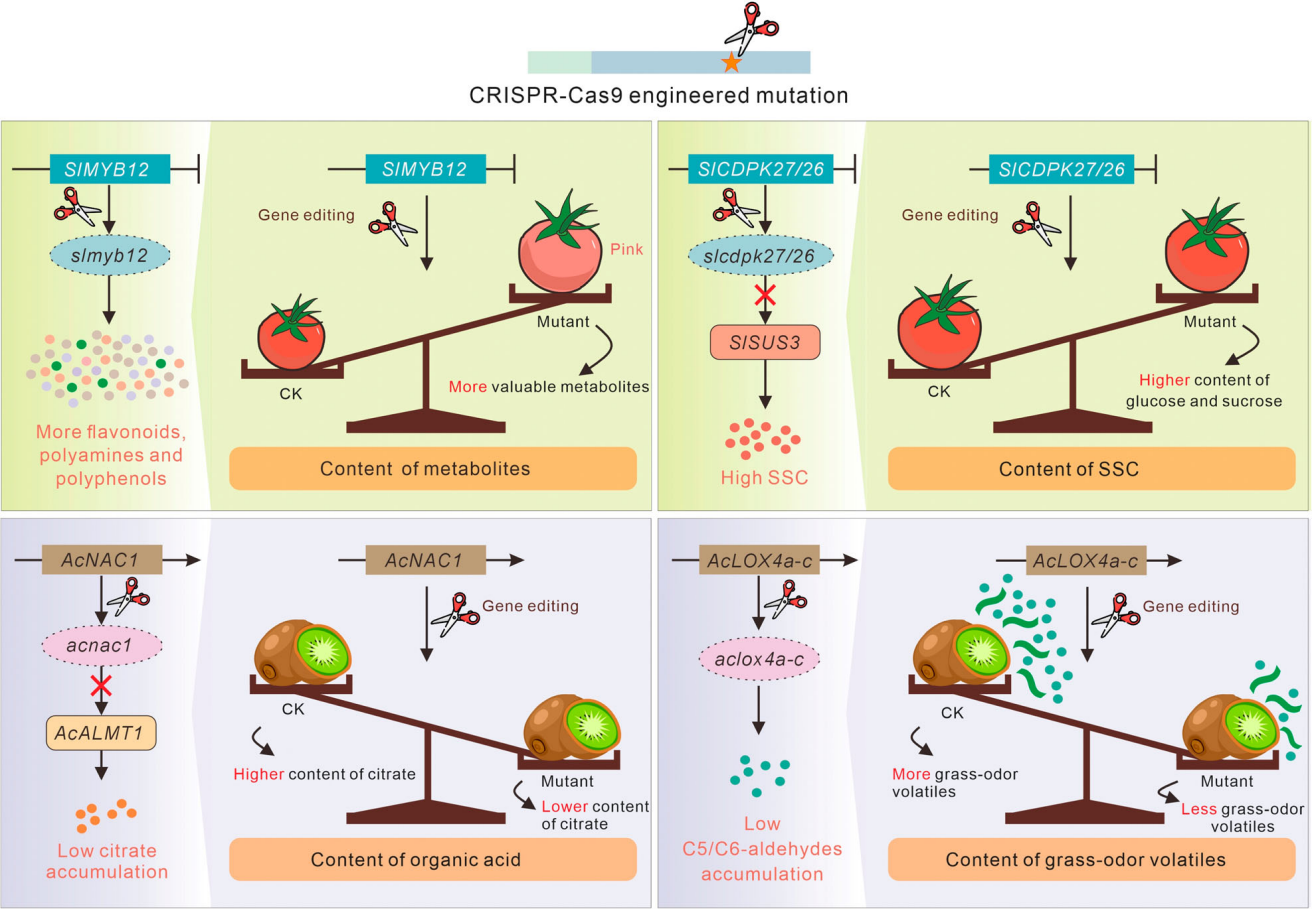
Case Studies in CRISPR/Cas9‐mediated plant flavor engineering An activation arrow (→) indicates positive transcriptional activation. Abbreviations: *Ac, Actinidia chinensis*; *ALMT1*, *Al‐ACTIVATED MALATE TRANSPORTER*; CDPK, calcium‐dependent protein kinase; CK, control; LOX, lipoxygenase; MYB, v‐myb avian myeloblastosis viral oncogene homolog; NAC, NAM/ATAF1/2/CUC; *Sl*, *Solanum lycopersicum*; SSC, soluble solids content; SUS, sucrose synthase.

## FUTURE PERSPECTIVES

Plant flavor remains difficult to improve because it is not a single metabolite trait, but a dynamic phenotype shaped by genomic variation, developmental stage, postharvest processes, and strong genotype‐by‐environment interactions. Although many loci, metabolites, and candidate pathways have been identified, the mechanisms linking natural genomic variation to stable flavor phenotypes remain only partly resolved. The next stage of research should therefore move beyond cataloging candidate loci and instead ask which variants are causal, how they alter flavor chemistry, and whether their effects persist across biological and environmental contexts.

A major obstacle is that many flavor‐associated alleles are regulatory, rare, or structurally complex, and are therefore poorly captured by single‐reference analysis (Zhu et al., 2019). This is especially relevant for flavor traits, because promoter variation, copy number variation, and gene presence‐absence variation can substantially alter metabolite accumulation without changing coding sequences. Tomato provides clear proof of principle. Pan‐genome analysis uncovered a rare *TomLoxC* promoter allele associated with apocarotenoid production and desirable fruit flavor, whereas graph pan‐genome analysis showed that incorporating structural variation improved genome‐to‐trait resolution and increased the estimated heritability of metabolite traits (Gao et al., 2019; Zhou et al., 2022). These findings suggest that future studies should routinely combine pan‐genome resources, haplotype‐based analysis, and systematic evaluation of elite cultivars, landraces, and wild relatives.

Causal variant discovery, however, is only the starting point. Flavor compounds are synthesized, transported, stored, and degraded in highly specific cell types and developmental stages, yet this spatial and temporal structure remains poorly resolved in most crops. Single‐cell and spatial multi‐omics can help close this gap by identifying where key pathways are active, which cell populations contribute most to metabolite production, and when regulatory switches are engaged during development or ripening (Li et al., 2024b; Zhu et al., 2025). Furthermore, both genotype‐by‐environment interactions and artificial intelligence‐assisted breeding will be central to the next phase of flavor research. Environmental effects should no longer be treated as experimental noise, because a favorable allele is only valuable if its effect is maintained across production environments, storage conditions, and supply chains. At the same time, AI‐assisted breeding can help integrate genomic, transcriptomic, metabolomic, phenotypic, and environmental data to improve causal locus discovery and the prediction of flavor performance under different conditions (Ferrão et al., 2023). However, these efforts are still limited by fragmented datasets, inconsistent phenotyping standards, and the weak connection between molecular measurements and consumer‐relevant flavor outcomes. The field, therefore, needs better‐linked datasets, clearer trait definitions, and more standardized pipelines that integrate chemical, sensory, and environmental information. In parallel, precision gene editing should extend beyond simple knockouts to promoter engineering, allele replacement, and other cis‐regulatory strategies that better recapitulate favorable natural variation while reducing trade‐offs in yield, shelf life, and stress tolerance.

Taken together, plant flavor research now requires a roadmap that moves from discovery to deployment (Figure 3). Multi‐omics integration should be used to identify causal genomic variants and define their effects on flavor‐related pathways. Artificial intelligence can then connect these datasets to improve causal inference and predict favorable alleles or haplotypes. Finally, gene editing, artificial intelligence, and synthetic biology can translate this knowledge into targeted crop improvement. This framework shifts the field from descriptive association to mechanism‐guided breeding and positions plant flavor as a tractable target for improving quality, nutrition, and sustainability.

**Figure 3:**
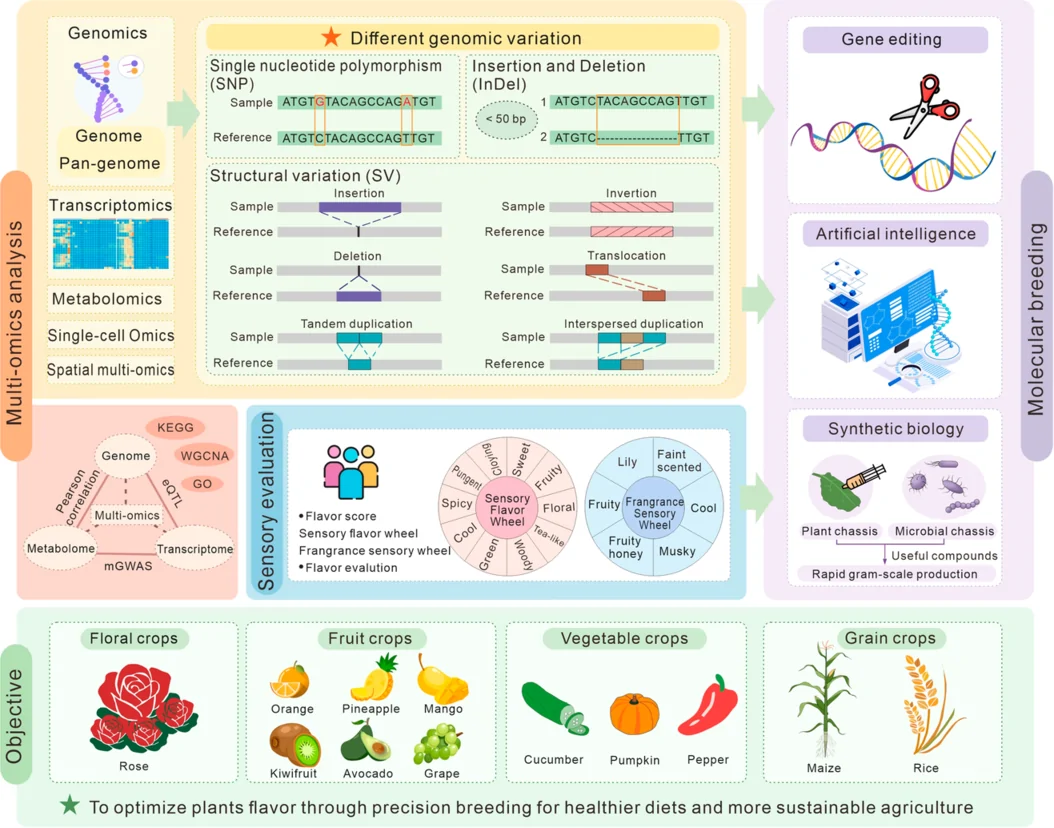
Integrative multi‐omics approaches to optimize flavor profiles in plants eQTL, expression quantitative trait loci; GO, gene ontology; KEGG, Kyoto Encyclopedia of Genes and Genomes; mGWAS, metabolite genome‐wide association study; WGCNA, weighted gene co‐expression network analysis.

## CONFLICTS OF INTEREST

The authors declare no conflicts of interest.

## AUTHOR CONTRIBUTIONS

H.H. drafted and revised the manuscript, generated the figures, and managed the tables and references. Y.H., Y.Z., and X.Z. revised certain parts of the main text. Y.H., R.X., and P.L. provided support for the project and revised the manuscript. All authors read and approved the final manuscript.
